# Effect of efgartigimod on muscle group subdomains in participants with generalized myasthenia gravis: post hoc analyses of the phase 3 pivotal ADAPT study

**DOI:** 10.1111/ene.16098

**Published:** 2023-10-16

**Authors:** Vera Bril, James F. Howard, Chafic Karam, Jan L. De Bleecker, Hiroyuki Murai, Kimiaki Utsugisawa, Peter Ulrichts, Edward Brauer, Sihui Zhao, Renato Mantegazza, Tuan Vu, the ADAPT Study Group

**Affiliations:** ^1^ Ellen and Martin Prosserman Centre for Neuromuscular Diseases University Health Network Toronto Ontario Canada; ^2^ University of Toronto Toronto Ontario Canada; ^3^ Department of Neurology University of North Carolina at Chapel Hill Chapel Hill North Carolina USA; ^4^ Penn Neuroscience Center–Neurology Hospital of the University of Pennsylvania Pennsylvania Philadelphia USA; ^5^ Department of Neurology Ghent University Hospital Ghent Belgium; ^6^ Department of Neurology, School of Medicine International University of Health and Welfare Tokyo Japan; ^7^ Department of Neurology Hanamaki General Hospital Hanamaki Japan; ^8^ argenx Ghent Belgium; ^9^ Department of Neuroimmunology and Neuromuscular Diseases Fondazione IRCCS Istituto Neurologico Carlo Besta Milan Italy; ^10^ Department of Neurology University of South Florida Morsani College of Medicine Tampa Florida USA

**Keywords:** ADAPT, efgartigimod, generalized myasthenia gravis, immunoglobulin G, neonatal Fc receptor

## Abstract

**Background and purpose:**

Generalized myasthenia gravis (gMG) is a rare, chronic, neuromuscular autoimmune disease mediated by pathogenic immunoglobulin G (IgG) autoantibodies. Patients with gMG experience debilitating muscle weakness, resulting in impaired mobility, speech, swallowing, vision and respiratory function. Efgartigimod is a human IgG1 antibody Fc fragment engineered for increased binding affinity to neonatal Fc receptor. The neonatal Fc receptor blockade by efgartigimod competitively inhibits endogenous IgG binding, leading to decreased IgG recycling and increased degradation resulting in lower IgG concentration.

**Methods:**

The safety and efficacy of efgartigimod were evaluated in the ADAPT study. Key efficacy outcome measures included Myasthenia Gravis Activities of Daily Living (MG‐ADL) and Quantitative Myasthenia Gravis (QMG) scores. Efgartigimod demonstrated significant improvement in both the MG‐ADL and QMG scores. This post hoc analysis aimed to determine whether all subdomains of MG‐ADL and QMG improved with efgartigimod treatment. Individual items of MG‐ADL and QMG were grouped into four subdomains: bulbar, ocular, limb/gross motor and respiratory. Change from baseline over 10 weeks in each subdomain was calculated for each group.

**Results:**

Greater improvements from baseline were seen across MG‐ADL subdomains in participants treated with efgartigimod compared with placebo. These improvements were typically observed 1 to 2 weeks after the first infusion and correlated with reductions in IgG. Similar results were observed across most QMG subdomains.

**Conclusions:**

These post hoc analyses of MG‐ADL and QMG subdomain data from ADAPT suggest that efgartigimod is beneficial in improving muscle function and strength across all muscle groups, leading to the observed efficacy in participants with gMG.

## INTRODUCTION

Generalized myasthenia gravis (gMG) is a rare, chronic, neuromuscular autoimmune disease mediated by pathogenic immunoglobulin G (IgG) autoantibodies that attack components of the postsynaptic membrane of the neuromuscular junction and impair neuromuscular transmission [1, 2, 3]. gMG significantly affects patients' quality of life and capacity to perform daily activities due to exertional fatigue and fluctuating weakness of bulbar, limb, axial, extraocular and respiratory muscles [1, 3]. Pathogenic IgG autoantibodies targeting the acetylcholine receptor (AChR) are detected in approximately 85% of individuals with myasthenia gravis (MG), whilst IgG antibodies directed against other components of the neuromuscular junction, such as muscle‐specific tyrosine kinase (MuSK) and low‐density lipoprotein receptor related protein 4, are identified in approximately 6% and 2% of patients with MG, respectively [4]. Treatment approaches include acetylcholinesterase inhibitors (AChEIs), corticosteroids, nonsteroidal immunosuppressive therapies (NSISTs), complement protein 5 inhibitors, thymectomy, intravenous immunoglobulin (IVIg) and plasma exchange (PLEX) [1, 5]. Although conventional treatment has resulted in substantial reductions in morbidity and mortality, 10% of patients are treatment refractory, and in up to 80% of patients complete remission is not achieved [6]. Development of new targeted therapies that reduce IgG autoantibodies may provide improved efficacy and tolerability [7].

The neonatal Fc receptor (FcRn) is responsible for IgG recycling, extending the half‐life of all IgG antibodies, and plays a role in IgG transcytosis and albumin recycling [8, 9]. Efgartigimod is a novel human IgG1 Fc fragment, a natural ligand of FcRn, engineered for increased binding affinity to FcRn whilst retaining the characteristic pH‐dependent binding of IgG–FcRn interactions [10, 11]. Efgartigimod competitively blocks endogenous IgG binding, preventing IgG recycling and increasing degradation of all IgG subtypes (including pathogenic IgG antibodies) without affecting IgG production or reducing other immunoglobulin isotypes (i.e., IgM, IgA, IgE, IgD) [10, 11, 12]. Efgartigimod has also been shown not to reduce serum albumin or to increase cholesterol levels [7, 10, 13].

Safety and efficacy of efgartigimod in participants with gMG were evaluated in an international, multicentre, randomized, placebo‐controlled phase 3 trial (ADAPT; NCT03669588), where efgartigimod was administered in treatment cycles of 4 weekly infusions [7]. The pivotal ADAPT trial was the basis for approval of efgartigimod for the treatment of adults with AChR antibody‐positive (AChR‐Ab+) gMG in the United States and the European Union and in Japan regardless of antibody status [14, 15]. The results of ADAPT have been previously described [7]. Briefly, ADAPT met the primary end‐point, where a significantly greater proportion of AChR‐Ab+ participants were Myasthenia Gravis Activities of Daily Living (MG‐ADL) responders during the first treatment cycle in the efgartigimod group (68%; *n* = 44/65) versus placebo (30%; *n* = 19/64; *p* < 0.0001) [7]. Similar results were observed in the overall population (AChR‐Ab+ and AChR‐Ab− participants), with 68% (*n* = 57/84) of participants treated with efgartigimod and 37% (*n* = 31/83) of participants receiving placebo being MG‐ADL responders (*p* < 0.0001) [7]. Likewise, a greater proportion of participants were Quantitative Myasthenia Gravis (QMG) responders during the first treatment cycle in the group treated with efgartigimod versus the group receiving placebo in both the AChR‐Ab+ (63% [41/65] vs. 14% [9/64]; *p* < 0.0001) and overall populations (*p* < 0.0001) [7]. Response in these analyses was defined as a ≥2‐point (MG‐ADL) or ≥3‐point (QMG) reduction in total score for ≥4 consecutive weeks, with the first improvement occurring by week 4 (1 week after the fourth infusion) of the cycle. Maximal decreases in MG‐ADL and QMG scores were observed at week 4 (1 week after the fourth infusion, at the time of maximal IgG reduction), with differences between treatment arms seen as early as 1 week after the first infusion in both the AChR‐Ab+ and overall populations [7]. Efgartigimod was well tolerated, with the most frequent adverse events being headache, nasopharyngitis, nausea, diarrhoea, upper respiratory tract infection and urinary tract infection.

Collectively, the results from ADAPT provided evidence of the safety and efficacy of efgartigimod in participants with gMG, including improvements in MG‐ADL and QMG scores. Importantly, the MG‐ADL and QMG are validated MG‐specific assessment tools composed of four subdomains representing the muscle groups affected by gMG: bulbar, ocular, limb/gross motor and respiratory (Table 1) [16, 17]. However, a previous study suggested that certain gMG treatments may have differential effects on various muscle subdomains. Specifically, the ocular subdomain scores of the Myasthenia Gravis Impairment Index changed more with prednisone than with IVIg or PLEX treatment, whilst the generalized subdomain scores changed more with IVIg and PLEX than with prednisone treatment [18]. Therefore, the objectives of the current post hoc analysis were to investigate whether the net clinical improvements in MG‐ADL and QMG total scores attributable to efgartigimod in the ADAPT study resulted from improvements across all subdomains and to evaluate the relative contributions of improvements in each subdomain to the observed treatment response to efgartigimod.

**TABLE 1:** MG‐ADL and QMG subdomains [16, 17].

Assessment	Subdomain				
	Ocular	Bulbar	Limb/gross motor	Respiratory	
MG‐ADL	Diplopia	Speech/voice	Ability to brush teeth or comb hair	Breathing	
	Ptosis	Swallowing	Ability to arise from chair		
		Chewing			
Possible scores	0–6	0–9	0–6	0–3^a^	Total 0–24
QMG	Diplopia (on primary or lateral gaze)	Speech/voice (onset of dysarthria following counting aloud from 1 to 50)	Hand grip strength		
	Ptosis (on upward gaze)	Swallowing (4 oz [~120 mL] water)	Limb strength (arm outstretched, 90°, sitting; leg outstretched, 45%–50%, supine)	Forced vital capacity	
	Facial muscles		Head lift (45%, supine)		
Possible scores	0–9	0–6	0–21	0–3	Total 0–39

Abbreviations: MG‐ADL, Myasthenia Gravis Activities of Daily Living; MGFA, Myasthenia Gravis Foundation of America; QMG, Quantitative Myasthenia Gravis.

^a^ ADAPT excluded participants requiring ventilatory assistance and intubation (MGFA class V), so the maximum possible score in the MG‐ADL respiratory subdomain during the ADAPT study was 2 points.

## METHOD

### Study design

A detailed methodology of ADAPT (NCT03669588) has previously been described [7]. Briefly, the study enrolled AChR‐Ab+ and AChR‐Ab− adults with gMG who were Myasthenia Gravis Foundation of America (MGFA) classes II−IV, had a baseline MG‐ADL total score of ≥5 (with >50% of the total score due to non‐ocular symptoms) and were on a stable dose of at least one oral gMG treatment (i.e., AChEIs, corticosteroids or NSISTs). Enrolled participants were randomized in a 1:1 ratio to efgartigimod or placebo for 26 weeks. All participants received an initial treatment cycle consisting of four weekly infusions of either efgartigimod (10 mg/kg) or matching placebo, with administration of subsequent cycles based on individual clinical evaluation, no sooner than 8 weeks after the start of the previous cycle (i.e., 5 weeks after the last infusion of the previous cycle). All participants provided written informed consent prior to the start of the study. International review boards and independent ethics committees provided written approval of the ADAPT protocol and all amendments. The trial was conducted according to the principles outlined in the Declaration of Helsinki.

### Efficacy assessments

#### Myasthenia Gravis Activities of Daily Living

Myasthenia Gravis Activities of Daily Living is a validated patient‐reported scale that assesses symptoms and ability to perform activities of daily living in patients with gMG within four muscle group subdomains (bulbar, ocular, limb/gross motor and respiratory) [16, 17]. Each item is scored on a scale from 0 to 3, with 0 representing normal function or absence of the corresponding symptom, and total scores range from 0 to 24 [16, 17]. A reduction of ≥2 points in MG‐ADL total score corresponds to clinically meaningful improvement [19, 20].

#### Quantitative Myasthenia Gravis

Quantitative Myasthenia Gravis is a validated, 13‐item, physician‐administered scale that provides an objective assessment of disease severity [21]. QMG measures strength and fatiguability in the same muscle groups as MG‐ADL, using objective measures of dysarthria, dysphagia, diplopia, ptosis and strength in facial, proximal limb, hand, neck and respiratory muscles [21]. A score of 0 (no symptoms) to 3 (severe symptoms) is assigned to each item; total score ranges from 0 to 39 [16, 21]. A change in total score of ≥3 points represents a clinically meaningful improvement [21], although this threshold may vary depending on baseline score [22]. Scoring of both MG‐ADL and QMG was performed by a trained and certified evaluator and assessed at each study visit.

### Subdomains

Individual items of MG‐ADL and QMG were grouped by subdomain (Table 1) [16, 17]. The ocular subdomain includes diplopia and ptosis items from MG‐ADL (possible score range 0–6) and diplopia (on primary or lateral gaze), ptosis (on upward gaze) and strength of eyelid closure (orbicularis oculi) items on QMG (possible score range 0–9). The bulbar subdomain includes speech/voice, swallowing and chewing items from MG‐ADL (possible score range 0–9) and speech/voice and swallowing items from QMG (possible score range 0–6). The limb/gross motor subdomain includes ability to brush teeth or comb hair and ability to arise from chair from MG‐ADL (possible score range 0–6) and grip strength, limb strength and head lift from QMG (possible score range 0–21). The respiratory subdomain comprises the breathing component from MG‐ADL (possible score range 0–3) and the forced vital capacity component from QMG (possible score range 0–3). ADAPT excluded participants requiring ventilatory assistance and intubation (MGFA class V), so the maximum possible MG‐ADL score in the respiratory subdomain during enrolment into the ADAPT study was 2 points.

### Statistical analysis

All efficacy analyses were performed in the modified intent‐to‐treat population, including all randomized participants with a recorded baseline MG‐ADL total score and at least one post‐baseline score. The current analyses consisted of a post hoc assessment of improvements in MG‐ADL and QMG subdomains in cycles 1 and 2 in participants with a baseline score of >0 (participants with no involvement in a particular subdomain cannot show improvement in that subdomain) in the AChR‐Ab+, AChR‐Ab− and overall populations. Actual scores and change from baseline in each subdomain were summarized using descriptive statistics for each treatment group in cycles 1 and 2 of ADAPT. Percentage change from baseline was also used due to the different ranges across subdomain scores. The two‐sample *t* test was used to calculate *p* values for change from baseline between treatment groups at each post‐baseline visit. The analyses are limited to baseline through week 10 owing to the very limited number of participants with available MG‐ADL and QMG measurements after week 10 (as participants had initiated a subsequent cycle of treatment).

## RESULTS

### Participants

Of 167 randomized participants, 129 (77%) were AChR‐Ab+ and 38 (23%) were AChR‐Ab– (of whom six [4%] were anti‐MuSK‐Ab+). Participant characteristics were representative of the general population of patients with gMG (Table 2). Most participants (86%; *n* = 144/167) were receiving immunosuppressive treatment (corticosteroids or NSISTs) at baseline. Baseline mean MG‐ADL (between 8.6 and 9.8 out of 24 across the different populations and treatment groups) and QMG (between 15.2 and 16.6 out of 39 across different populations and treatment groups) total scores suggested considerable disease burden despite stable concomitant gMG treatment (Table 2). The proportions of participants with baseline involvement in bulbar, limb/gross motor, ocular and respiratory subdomains, as well as the mean baseline scores in these subdomains, are detailed in Table 3 for AChR‐Ab+ participants, in Table S1 for the overall population and in Table S2 for AChR‐Ab− participants.

**TABLE 2:** Baseline characteristics.

Characteristic	Overall population		AChR‐Ab+ participants		AChR‐Ab− participants	
	Efgartigimod (*n* = 84)	Placebo (*n* = 83)	Efgartigimod (*n* = 65)	Placebo (*n* = 64)	Efgartigimod (*n* = 19)	Placebo (*n* = 19)
Age, mean (SD), years	45.9 (14.4)	48.2 (15.0)	44.7 (15.0)	49.2 (15.5)	50.2 (11.6)	44.8 (12.6)
Sex, female, *n* (%)	63 (75)	55 (66)	46 (71)	40 (63)	17 (90)	15 (79)
Time since diagnosis, mean (SD), years	10.1 (9.0)	8.8 (7.6)	9.7 (8.3)	8.9 (8.2)	11.7 (11.5)	8.5 (5.2)
Total MG‐ADL score, mean (SD)	9.2 (2.6)	8.8 (2.3)	9.0 (2.5)	8.6 (2.1)	9.7 (3.1)	9.8 (2.5)
Total QMG score, mean (SD)	16.2 (5.0)	15.5 (4.6)	16.0 (5.1)	15.2 (4.4)	16.6 (4.6)	16.5 (5.2)
MGFA class at screening, *n* (%)						
Class II	34 (40)	31 (37)	28 (43)	25 (39)	6 (32)	6 (32)
Class III	47 (56)	49 (59)	35 (54)	36 (56)	12 (63)	13 (68)
Class IV	3 (4)	3 (4)	2 (3)	3 (5)	1 (5)	0 (0)
Prior treatment with NSIST, *n* (%)	62 (74)	57 (69)	47 (72)	43 (67)	15 (79)	14 (74)
MG therapies at baseline, *n* (%)						
Any AChEI	71 (85)	67 (81)	57 (88)	57 (89)	14 (74)	10 (53)
Any steroid	60 (71)	67 (81)	46 (71)	51 (80)	14 (74)	16 (84)
Any NSIST	51 (61)	51 (61)	40 (62)	37 (58)	11 (58)	14 (74)
Steroid and NSIST	43 (51)	44 (53)	34 (52)	31 (48)	9 (47)	13 (68)
No steroid or NSIST (AChEI only)	16 (19)	7 (8)	13 (20)	6 (9)	3 (16)	1 (5)

Abbreviations: AChEI, acetylcholinesterase inhibitor; AChR‐Ab, anti‐acetylcholine receptor antibody; MG, myasthenia gravis; MG‐ADL, Myasthenia Gravis Activities of Daily Living; MGFA, Myasthenia Gravis Foundation of America; NSIST, nonsteroidal immunosuppressive therapy; QMG, Quantitative Myasthenia Gravis.

**TABLE 3:** Disease activity in MG‐ADL and QMG subdomains at cycle baseline in AChR‐Ab+ participants.

Assessment	Treatment (total *N* at baseline)	Subdomain^a^							
		Ocular		Bulbar		Limb/gross motor		Respiratory	
MG‐ADL		*n* (%)	Mean (SE) (range 1–6)	*n* (%)	Mean (SE) (range 1–9)	*n* (%)	Mean (SE) (range 1–6)	*n* (%)	Mean (SE) (range 1–2)^b^
Cycle 1	Efgartigimod (*N* = 65)	58 (89)	2.64 (0.17)	64 (98)	3.16 (0.16)	60 (92)	2.77 (0.13)	56 (86)	1.18 (0.05)
	Placebo (*N* = 64)	54 (84)	2.35 (0.15)	64 (100)	2.88 (0.14)	61 (95)	2.74 (0.14)	58 (91)	1.21 (0.05)
Cycle 2	Efgartigimod (*N* = 51)	47 (92)	3.04 (0.19)	50 (98)	3.22 (0.21)	49 (96)	2.76 (0.15)	45 (88)	1.47 (0.08)
	Placebo (*N* = 43)	37 (86)	2.84 (0.23)	42 (98)	2.90 (0.20)	42 (98)	2.83 (0.16)	35 (81)	1.26 (0.07)
QMG		*n* (%)	Mean (SE) (range 1–9)	*n* (%)	Mean (SE) (range 1–6)	*n* (%)	Mean (SE) (range 1–21)	*n* (%)	Mean (SE) (range 1–3)
Cycle 1	Efgartigimod (*N* = 65)	62 (95)	4.02 (0.27)	51 (78)	2.16 (0.14)	65 (100)	10.03 (0.39)	22 (34)	1.41 (0.14)
	Placebo (*N* = 64)	61 (95)	3.56 (0.21)	40 (63)	1.85 (0.15)	62 (97)	9.95 (0.39)	25 (39)	1.48 (0.14)
Cycle 2	Efgartigimod (*N* = 51)	47 (92)	4.00 (0.28)	32 (63)	2.47 (0.26)	51 (100)	9.45 (0.48)	23 (45)	1.30 (0.13)
	Placebo (*N* = 43)	42 (98)	3.95 (0.27)	27 (63)	2.07 (0.25)	43 (100)	10.65 (0.42)	23 (53)	1.48 (0.16)

Abbreviations: MG‐ADL, Myasthenia Gravis Activities of Daily Living; MGFA, Myasthenia Gravis Foundation of America; QMG, Quantitative Myasthenia Gravis; SE, standard error.

^a^ Only participants with a baseline score of >0 in each subdomain were included in the analysis.

^b^ ADAPT excluded participants requiring ventilatory assistance and intubation (MGFA class V), so the maximum possible score in the MG‐ADL respiratory subdomain during the ADAPT study was 2 points.

### Myasthenia Gravis Activities of Daily Living subdomains analysis

**FIGURE 1:**
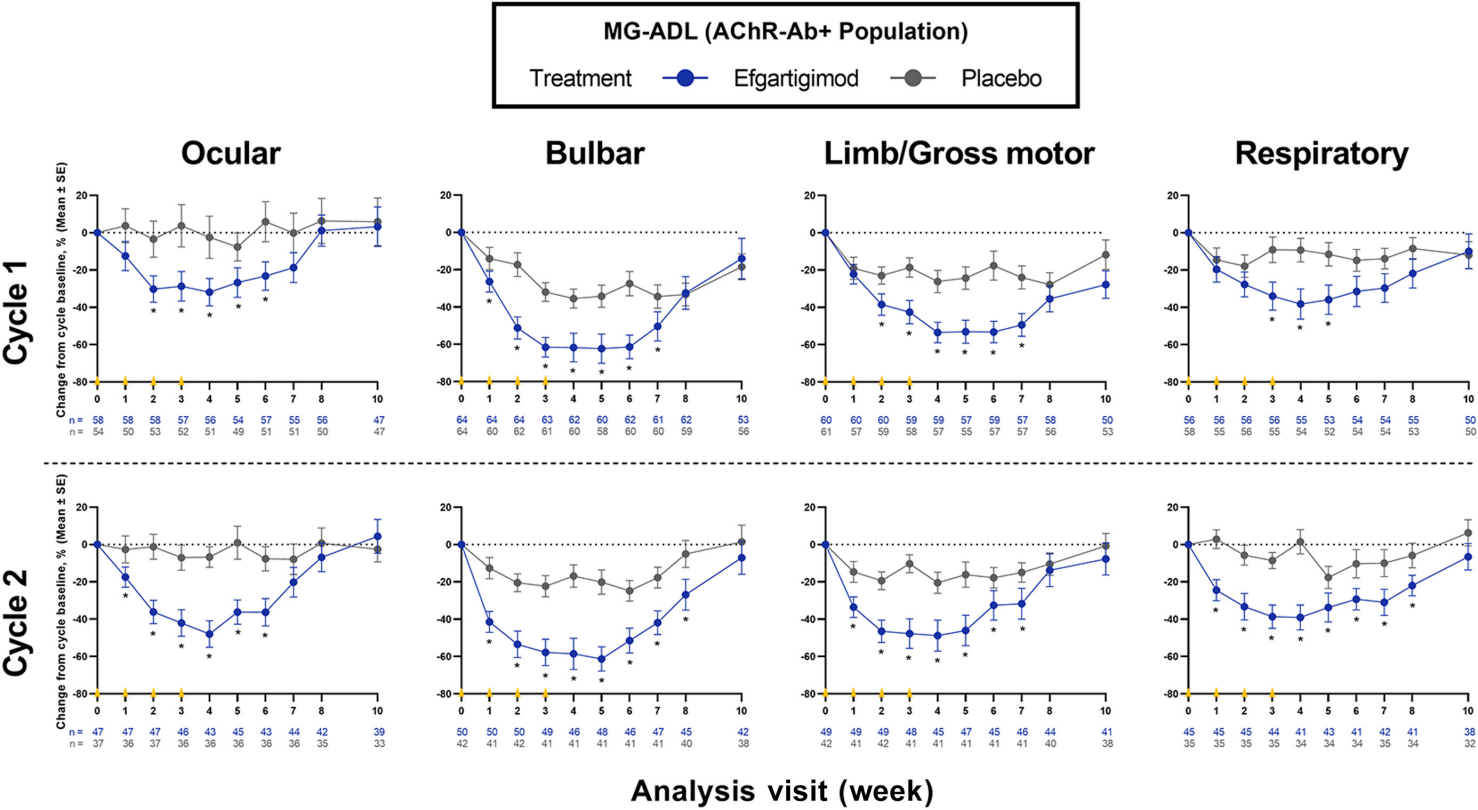
Percentage change from baseline in MG‐ADL subdomains over 10 weeks across cycles 1 and 2 in AChR‐Ab+ participants. Differences between treatment arms were noted across most MG‐ADL subdomains as early as 1–2 weeks after treatment. Each cycle consisted of 4 weekly infusions occurring at weeks 0, 1, 2 and 3 (yellow triangles) of either efgartigimod (10 mg/kg) or matching placebo. AChR‐Ab+, acetylcholine receptor antibody positive; MG‐ADL, Myasthenia Gravis Activities of Daily Living; SE, standard error. *p < 0.05 (two‐sample *t* test).

Significantly greater improvements from baseline were seen in AChR‐Ab+ participants treated with efgartigimod in all MG‐ADL subdomains in both cycles 1 and 2 compared with placebo (Figure 1). Differences between participants treated with efgartigimod versus those receiving placebo were noted across most MG‐ADL subdomains as early as 1 to 2 weeks after treatment initiation. In AChR‐Ab+ participants, mean (SE) score changes from baseline to week 4 (1 week after the fourth infusion) in participants receiving efgartigimod versus placebo in each MG‐ADL subdomain were ocular, −0.82 (0.17) versus −0.18 (0.17) (*p* = 0.0088); bulbar, −2.10 (0.21) versus −0.88 (0.14) (*p* < 0.0001); limb/gross motor, −1.49 (0.16) versus −0.70 (0.15) (*p* = 0.0006); and respiratory, −0.49 (0.10) versus −0.15 (0.08) (*p* = 0.0065) in cycle 1. Similarly, mean (SE) MG‐ADL subdomain score changes in cycle 2 were ocular, −1.49 (0.21) versus −0.25 (0.13) (*p* < 0.0001); bulbar, −2.13 (0.29) versus −0.51 (0.16) (*p* < 0.0001); limb/gross motor, −1.24 (0.17) versus −0.49 (0.12) (*p* = 0.0006); and respiratory, −0.61 (0.10) versus −0.03 (0.08) (*p* < 0.0001). Results of the MG‐ADL subdomains analysis in the overall and AChR‐Ab− populations are reported in the Supporting Information (Appendix S1 and Figures S1 and S3, respectively).

### Quantitative Myasthenia Gravis subdomains analysis

**FIGURE 2:**
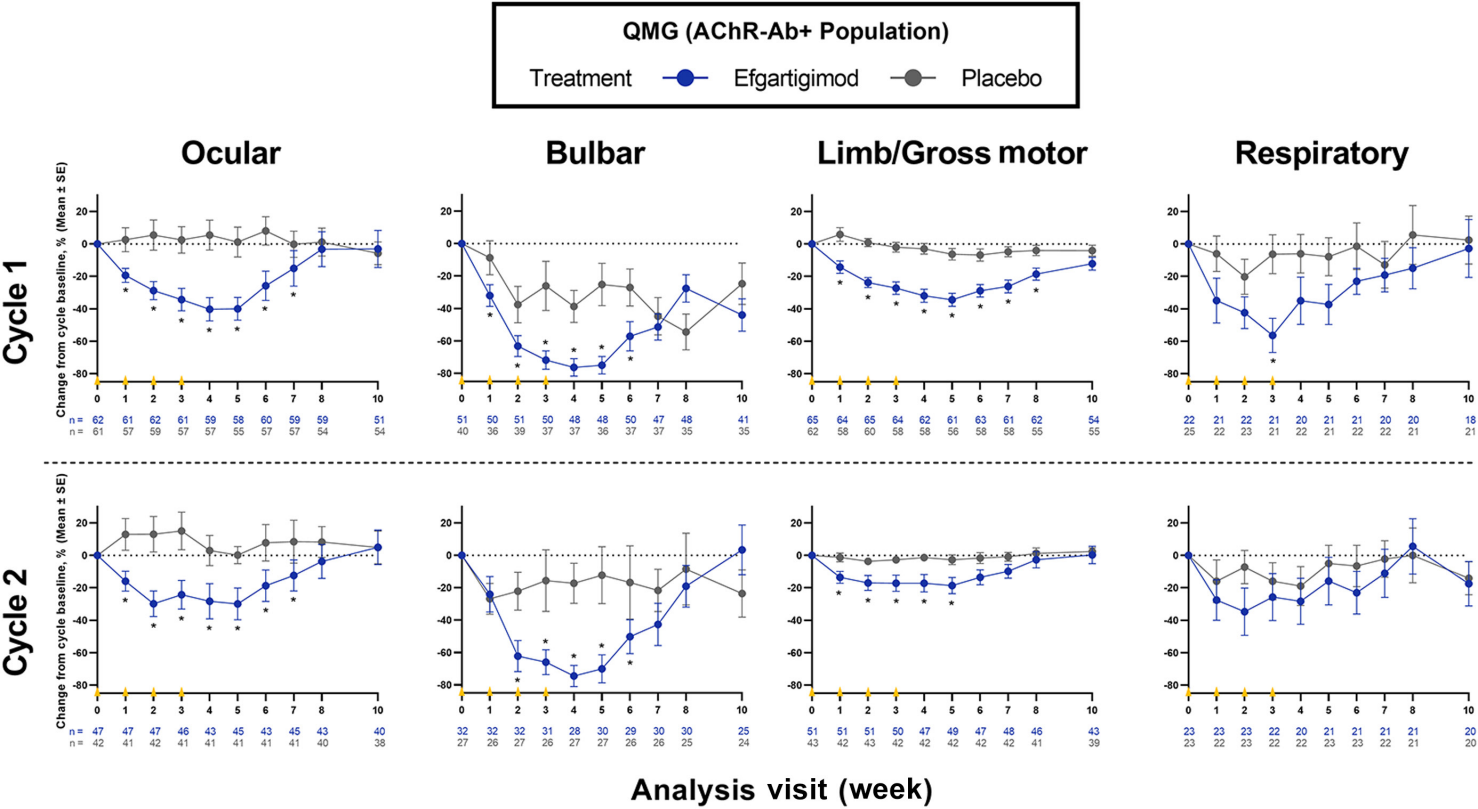
Percentage change from baseline in QMG subdomains over 10 weeks across cycles 1 and 2 in AChR‐Ab+ participants. As with MG‐ADL, differences between treatment arms were noted across most QMG subdomains as early as 1–2 weeks after treatment. Each cycle consisted of 4 weekly infusions occurring at weeks 0, 1, 2 and 3 (yellow triangles) of either efgartigimod (10 mg/kg) or matching placebo. AChR‐Ab+, acetylcholine receptor antibody positive; MG‐ADL, Myasthenia Gravis Activities of Daily Living; QMG, Quantitative Myasthenia Gravis; SE, standard error. *p < 0.05 (two‐sample *t* test).

In AChR‐Ab+ participants treated with efgartigimod, significantly greater improvements from baseline were observed compared with placebo for all QMG subdomains except respiratory in both cycles 1 and 2 (Figure 2). Differences between treatment arms in most QMG subdomains were observed as early as 1 to 2 weeks after treatment initiation. In cycle 1, mean (SE) QMG subdomain score changes from baseline to week 4 (1 week after the fourth infusion) in AChR‐Ab+ participants treated with efgartigimod versus receiving placebo in each subdomain were ocular, −1.80 (0.24) versus −0.16 (0.17) (*p* < 0.0001); bulbar, −1.69 (0.17) versus −0.78 (0.18) (*p* = 0.0004); limb/gross motor, −3.13 (0.43) versus −0.40 (0.25) (*p* < 0.0001); and respiratory, −0.60 (0.21) versus −0.23 (0.17) (*p* = 0.1793). Similarly, mean (SE) QMG subdomain score changes in cycle 2 were ocular, −1.60 (0.33) versus −0.17 (0.20) (*p* = 0.0004); bulbar, −2.04 (0.28) versus −0.59 (0.24) (*p* = 0.0003); limb/gross motor, −1.77 (0.48) versus −0.33 (0.23) (*p* = 0.0085); and respiratory, −0.45 (0.18) versus −0.27 (0.13) (*p* = 0.4429). See the Supporting Information (Appendix S1 and Figures S2 and S4) for results of the QMG subdomains analysis in the overall and AChR‐Ab− populations, respectively.

## DISCUSSION

In the ADAPT study, efgartigimod was shown to be efficacious and well tolerated in participants with gMG. Amongst participants who were AChR‐Ab+, 68% of those treated with efgartigimod (vs. 30% placebo; *p* < 0.0001) were MG‐ADL responders in cycle 1. Likewise, 63% of AChR‐Ab+ participants in the efgartigimod group (vs. 14% placebo; *p* < 0.0001) were QMG responders in cycle 1 [7]. This exploratory post hoc analysis of MG‐ADL and QMG subdomains demonstrated that all subdomains contributed to the net improvements in composite scores experienced by participants treated with efgartigimod during ADAPT. Participants treated with efgartigimod showed significant improvements from baseline across all subdomains (ocular, bulbar, limb/gross motor and respiratory function) of the MG‐ADL score. Similar observations, with significant improvements in participants treated with efgartigimod (vs. placebo), were found in the physician‐reported QMG score subdomains of ocular, bulbar and limb/gross motor functions along with numerically greater improvements in the respiratory subdomain.

Improvements were observed within 1 to 2 weeks after treatment initiation across most MG‐ADL and QMG subdomains, which paralleled the time to improvement in the composite scores [7]. Whilst a previous study suggested that some treatments can exert a differential effect on individual subdomains of composite MG scores [18], the present post hoc analyses demonstrated that efgartigimod is efficacious across subdomains irrespective of whether the symptoms are patient reported (MG‐ADL) or physician assessed (QMG).

Differences between participants treated with efgartigimod versus receiving placebo were small in the respiratory subdomain for both MG‐ADL and QMG, with results reaching statistical significance for the MG‐ADL scale and a trend towards numerical improvement at most time points for QMG. Of note, the ADAPT study excluded participants requiring ventilatory assistance and intubation (MGFA class V) and therefore had a smaller maximum possible baseline score in the respiratory subdomain for both MG‐ADL and QMG. Moreover, at baseline, the number of participants available for evaluation (with a score >0) in the respiratory subdomain using the QMG scale was fewer than the number of participants available for evaluation with the MG‐ADL scale. Thus, the small sample size in the respiratory subdomain of the QMG score combined with its reduced sensitivity to measure any change in response in vital capacity (respiratory function) may have resulted in a numerical (as opposed to statistically significant) improvement in participants treated with efgartigimod versus receiving placebo.

Patient‐reported outcomes are becoming increasingly important for both regulatory authorities (for approval considerations) and payors (for considerations of treatment utilization) [21, 23]. With the emphasis shifting toward patient‐reported outcomes, several tools sensitive to the domains of ocular, bulbar or generalized weakness have been developed [21, 24]. The patient's perspective of gMG is important [23], and thus utilization of a patient‐reported scale (e.g., MG‐ADL) provides valuable information. The findings on subdomains of the MG‐ADL scale from this exploratory analysis further indicate the significant improvements of participants receiving efgartigimod versus receiving placebo.

There are several important limitations to the present analysis. Only total MG‐ADL/QMG scores have been validated as outcome measures, whilst the individual subdomains have not been independently validated. Additionally, this is a subgroup post hoc analysis, and statistical results may therefore be affected by multiplicity. Another limitation is the small sample size, especially in subdomains with low baseline involvement, which decreases the power of statistical tests. Finally, the ADAPT trial excluded patients with purely ocular (i.e., those with MGFA class I disease) or predominantly ocular symptoms (requiring a baseline MG‐ADL total score ≥5 with >50% of total score attributable to non‐ocular symptoms) and therefore this analysis does not describe the full extent to which efgartigimod would affect these patients; however, as described above, efgartigimod significantly improved ocular symptoms as assessed by both the MG‐ADL and QMG in patients where ocular involvement occurred in the setting of generalized disease.

These results add to findings of the pivotal ADAPT study in which efgartigimod demonstrated significant and repeatable clinical benefit as measured by total MG‐ADL and QMG scores. The current findings confirm that efgartigimod can improve function and strength across all muscle groups involved in the symptomatology of gMG, with potential implications in reducing disease burden in patients affected by weakness in these subdomains. These data further support the benefit efgartigimod offers, across MG‐ADL and QMG subdomains, in a broad population of patients with gMG.

## AUTHOR CONTRIBUTIONS

**Vera Bril:** Conceptualization; writing – review and editing; investigation. **James F. Howard Jr:** Conceptualization; writing – review and editing; investigation. **Chafic Karam:** Conceptualization; investigation; writing – review and editing. **Jan L. De Bleecker:** Conceptualization; writing – review and editing; investigation. **Hiroyuki Murai:** Conceptualization; investigation; writing – review and editing. **Kimiaki Utsugisawa:** Conceptualization; investigation; writing – review and editing. **Peter Ulrichts:** Conceptualization; methodology; writing – review and editing. **Edward Brauer:** Conceptualization; writing – review and editing; methodology. **Sihui Zhao:** Conceptualization; writing – review and editing; methodology; formal analysis. **Renato Mantegazza:** Conceptualization; writing – review and editing; investigation. **Tuan Vu:** Conceptualization; writing – review and editing; investigation.

## FUNDING INFORMATION

The ADAPT study was sponsored and funded by argenx.

## CONFLICT OF INTEREST STATEMENT

VB has received research support from AZ‐Alexion, Grifols, CSL, UCB, argenx, Takeda, Octapharma, Akcea, Momenta (J&J), Immunovant, Ionis and Viela. JFH has received research support (paid to his institution) from Alexion Pharmaceuticals Inc., argenx, Cartesian Therapeutics, the Centers for Disease Control and Prevention, Myasthenia Gravis Foundation of America, Muscular Dystrophy Association, National Institutes of Health (including the National Institute of Neurological Disorders and Stroke and the National Institute of Arthritis and Musculoskeletal and Skin Diseases), Patient‐Centered Outcomes Research Institute, Ra Pharmaceuticals Inc. (now UCB), honoraria from AcademicCME, Alexion Pharmaceuticals Inc., argenx, Biologix Pharma, F. Hoffman LaRoche Ltd, Horizon Therapeutics plc, Medscape CME, Merck EMB Serono, NMD Pharma, Novartis Pharmaceuticals, PeerView CME, Ra Pharmaceuticals Inc. (now UCB), Regeneron Pharmaceuticals Inc., Sanofi US and Zai Labs; and nonfinancial support from Alexion Pharmaceuticals Inc., argenx, Ra Pharmaceuticals Inc. (now UCB) and Toleranzia AB. CK served as a deputy editor for *Neurology* and as a consultant for Acceleron Pharma Inc., Akcea Therapeutics, Alnylam Pharmaceuticals Inc., argenx, Biogen, CSL Behring and Sanofi Genzyme. Dr Karam has received personal compensation for speaking engagements from Akcea Therapeutics, Alnylam Pharmaceuticals Inc., CSL Behring and Sanofi Genzyme and research/grant support from Akcea Therapeutics and Sanofi Genzyme. JLDB has served as a consultant for argenx, Alexion Pharmaceuticals Inc., CSL, UCB Pharma, Alnylam Pharmaceuticals Inc. and Sanofi Genzyme. HM has served as a paid consultant for Alexion, AstraZeneca Rare Disease, argenx, UCB Pharma and Roche; has received speaker honoraria from the Japan Blood Products Organization and Chugai Pharmaceutical; and has received research support from the Ministry of Health, Labour and Welfare, Japan. KU has served as a paid consultant for argenx, Ra Pharmaceuticals Inc., UCB Pharma, Janssen Pharma, Chugai Pharma, Merck and Mitsubishi Tanabe Pharma Corporation and has received speaker honoraria from argenx, Alexion Pharmaceuticals Inc., UCB Pharma and the Japan Blood Products Organization. PU, EB and SZ are employees of argenx. RM has received consulting fees/honoraria or support for meeting participation from Alexion Pharmaceuticals, argenx, Ra Pharmaceuticals (now UCB), Biomarin, Catalyst, UCB, TEVA, Merck, Roche and Biogen. TV has served as a speaker for Alexion, argenx, CSL Behring and Allergan/AbbVie. He has performed consulting work related to MG for argenx, Alexion/AstraZeneca and UCB, and participated in trials in MG sponsored by Alexion/AstraZeneca, argenx, Ra/UCB, Horizon/Viela Bio, Regeneron, Janssen/Momenta, Immunovant, Cartesians Therapeutics and Sanofi.

## Supporting information


Appendix S1

Figure S1

Figure S2

Figure S3

Figure S4

Table S1

Table S2

## Data Availability

argenx is committed to responsible data sharing regarding the clinical trials they fund. Included in this commitment is access to anonymized, individual and trial‐level data (analysis datasets) and other information (e.g., protocols and clinical study reports), as long as the trials are not part of an ongoing or planned regulatory submission. This includes requests for clinical trial data for unlicensed products and indications. These clinical trial data can be requested by qualified researchers who engage in rigorous independent scientific research and will only be provided after review and approval of a research proposal and statistical analysis plan and execution of a data sharing agreement. Data requests can be submitted at any time, and the data will be accessible for 12 months. Requests can be submitted to esr@argenx.com.
