# Supplementary material for: Effect of efgartigimod on muscle group subdomains in participants with generalized myasthenia gravis: post hoc analyses of the phase 3 pivotal ADAPT study
Source: Eur J Neurol. 2023 Oct 16;31(1):e16098. doi: 10.1111/ene.16098 (PMC11235734; doi:10.1111/ene.16098)

**SUPPORTING INFORMATION**

For the original article: **Effect of Efgartigimod on Muscle Group Subdomains in Participants With Generalized Myasthenia Gravis: Post Hoc Analyses of the ADAPT Study**

**Supplementary Results**

*MG-ADL Subdomains Analysis, Overall Population*

In the overall population, similar results favoring efgartigimod were seen compared with placebo (Figure S1), where mean (SE) MG-ADL score changes from baseline to Week 4 (1 week after the fourth infusion) in participants treated with efgartigimod versus those receiving placebo in the subdomains were: ocular, -0.91 (0.16) versus -0.31 (0.15, *P*=0.0069); bulbar, -1.96 (0.19) versus -0.94 (0.12, *P*<0.0001); limb/gross motor, -1.42 (0.14) versus -0.70 (0.13, *P*=0.0003); and respiratory, -0.49 (0.08) versus -0.20 (0.07, *P*=0.0084), in Cycle 1. Similarly, mean (SE) MG-ADL subdomain score changes in Cycle 2 were: ocular, -1.42 (0.19) versus -0.58 (0.20, *P*=0.0037); bulbar, -2.03 (0.25) versus -0.70 (0.15, *P*<0.0001); limb/gross motor, -1.25 (0.15) versus -0.65 (0.15, *P*=0.0055); and respiratory, -0.63 (0.10) versus -0.13 (0.08, *P*=0.0001).

*QMG Subdomains Analysis, Overall Population*

Results in the overall population similarly favored efgartigimod compared with placebo (Figure S2), where mean (SE) QMG score changes from baseline to Week 4 (1 week after the fourth infusion) in the subdomains were: ocular, -1.74 (0.22) versus -0.35 (0.17, *P*<0.0001); bulbar, -1.58 (0.15) versus -0.75 (0.16, *P*=0.0003); limb/gross motor, -3.25 (0.37) versus -1.03 (0.30, *P*<0.0001); and respiratory, -0.61 (0.19) versus -0.21 (0.16, *P*=0.1122), in Cycle 1. Similarly, mean (SE) QMG subdomain score changes in Cycle 2 were: ocular, -1.57 (0.29) versus -0.44 (0.23, *P*=0.0029); bulbar, -1.97 (0.25) versus -0.81 (0.21, *P*=0.0007); limb/gross motor, -1.81 (0.42) versus -0.76 (0.25, *P*=0.0343); and respiratory, -0.40 (0.15) versus -0.23 (0.13, *P*=0.3998), for participants treated with efgartigimod versus those receiving placebo.

*MG-ADL Subdomains Analysis, AChR-Ab- Population*

In AChR-Ab- participants, a numerical trend favoring efgartigimod were observed compared with placebo (Figure S3) across most subdomains; mean (SE) MG-ADL score changes from baseline to Week 4 (1 week after the fourth infusion) in participants treated with efgartigimod versus those receiving placebo in the subdomains were: ocular, -1.29 (0.40) versus -0.75 (0.31, *P*=0.2982); bulbar, -1.47 (0.39) versus -1.11 (0.26, *P*=0.4474); limb/gross motor, -1.18 (0.23) versus -0.71 (0.28, *P*=0.2054); and respiratory, -0.50 (0.16) versus -0.38 (0.18, *P*=0.6054), in Cycle 1. Similarly, mean (SE) MG-ADL subdomain score changes in Cycle 2 were: ocular, -1.10 (0.50) versus -1.58 (0.66, *P*=0.5661); bulbar, -1.67 (0.47) versus -1.33 (0.38, *P*=0.5836); limb/gross motor, -1.25 (0.35) versus -1.15 (0.45, *P*=0.8678); and respiratory, -0.73 (0.24) versus -0.42 (0.19, *P*=0.3219). In general, AChR-Ab- and AChR-Ab+ participants treated with efgartigimod exhibited similar MG-ADL responses, although a greater response to placebo was observed in the AChR-Ab- compared with AChR-Ab+ population.

*QMG Subdomains Analysis, AChR-Ab- Population*

Results in AChR-Ab- participants showed a similar numerical trend favoring efgartigimod compared with placebo across most subdomains (Figure S4), where mean (SE) QMG score changes from baseline to Week 4 (1 week after the fourth infusion) in the subdomains were: ocular, -1.53 (0.50) versus -0.94 (0.47, *P*=0.4027); bulbar, -1.09 (0.37) versus -0.64 (0.37, *P*=0.4010); limb/gross motor, -3.71 (0.72) versus -2.95 (0.85, *P*=0.5018); and respiratory, -0.67 (0.33) versus -0.17 (0.40, *P*=0.3721), in Cycle 1.

Similarly, mean (SE) QMG subdomain score changes in Cycle 2 were: ocular, -1.45 (0.65) versus -1.31 (0.67, *P*=0.8769); bulbar, -1.67 (0.61) versus -1.44 (0.38, *P*=0.7652); limb/gross motor, -2.00 (0.92) versus -2.15 (0.64, *P*=0.8922); and respiratory, -0.20 (0.20) versus 0.00 (0.41, *P*=0.6806), for participants treated with efgartigimod versus those receiving placebo. Similar to MG-ADL results, AChR-Ab- and AChR-Ab+ participants treated with efgartigimod exhibited similar QMG responses, with a larger placebo response in AChR-Ab- compared to AChR-Ab+ participants.

**Supporting Tables**

**Table S1. Disease Activity in MG-ADL and QMG Subdomains at Cycle Baseline in the Overall Population**

|  | Treatment (total N at baseline) | Subdomain^†^ | | | | | | | | |
| --- | --- | --- | --- | --- | --- | --- | --- | --- | --- | --- |
| Assessment |  | **Ocular** | | **Bulbar** | | **Limb/gross motor** | | | **Respiratory** | |
| MG-ADL | | **n (%)** | **Mean (SE)** (range 1-6) | **n (%)** | **Mean (SE)** (range 1-9) | **n (%)** | **Mean (SE)** (range 1-6) | | **n (%)** | **Mean (SE)** (range 1-2^‡^) |
| Cycle 1 | Efgartigimod (N=84) | 74 (88) | 2.84 (0.17) | 83 (99) | 3.12 (0.15) | 79 (94) | 2.73 (0.11) | 73 (87) | | 1.19 (0.05) |
|  | Placebo  (N=83) | 70 (84) | 2.64 (0.15) | 83 (100) | 2.87 (0.13) | 78 (94) | 2.83 (0.12) | 74 (89) | | 1.22 (0.05) |
| Cycle 2 | Efgartigimod (N=63) | 57 (90) | 3.05 (0.18) | 62 (98) | 3.19 (0.18) | 61 (97) | 2.79 (0.13) | 56 (89) | | 1.45 (0.07) |
|  | Placebo  (N=57) | 49 (86) | 3.08 (0.20) | 55 (96) | 3.00 (0.18) | 56 (98) | 2.91 (0.14) | 48 (84) | | 1.25 (0.06) |
| QMG | | **n (%)** | **Mean (SE)** (range 1-9) | **n (%)** | **Mean (SE)** (range 1-6) | **n (%)** | **Mean (SE)** (range 1-21) | | **n (%)** | **Mean (SE)** (range 1-3) |
| Cycle 1 | Efgartigimod (N=84) | 81 (96) | 4.10 (0.24) | 63 (75) | 2.06 (0.13) | 84 (100) | 10.20 (0.33) | | 27 (32) | 1.44 (0.12) |
|  | Placebo  (N=83) | 79 (95) | 3.73 (0.20) | 54 (65) | 1.87 (0.13) | 81 (98) | 10.07 (0.34) | | 31 (37) | 1.48 (0.13) |
| Cycle 2 | Efgartigimod (N=63) | 58 (92) | 4.14 (0.27) | 38 (60) | 2.39 (0.23) | 63 (100) | 9.33 (0.44) | | 28 (44) | 1.29 (0.11) |
|  | Placebo  (N=57) | 56 (98) | 4.09 (0.24) | 37 (65) | 2.08 (0.21) | 57 (100) | 10.51 (0.37) | | 28 (49) | 1.46 (0.15) |

^†^Only participants with a baseline score of >0 in each subdomain were included in the analysis. ^‡^ADAPT excluded participants requiring ventilatory assistance and intubation (MGFA Class V), so the maximum possible score in the MG-ADL respiratory subdomain during the ADAPT study was 2 points. MG-ADL, Myasthenia Gravis Activities of Daily Living; MGFA, Myasthenia Gravis Foundation of America; QMG, Quantitative Myasthenia Gravis; SE, standard error.

**Table S2. Disease Activity in MG-ADL and QMG Subdomains at Cycle Baseline in the AChR-Ab- Population**

|  | Treatment (total N at baseline) | Subdomain^†^ | | | | | | | | |
| --- | --- | --- | --- | --- | --- | --- | --- | --- | --- | --- |
| Assessment |  | **Ocular** | | **Bulbar** | | **Limb/gross motor** | | | **Respiratory** | |
| MG-ADL | | **n (%)** | **Mean (SE)** (range 1-6) | **n (%)** | **Mean (SE)** (range 1-9) | **n (%)** | **Mean (SE)** (range 1-6) | | **n (%)** | **Mean (SE)** (range 1-2^‡^) |
| Cycle 1 | Efgartigimod (N=19) | 16 (84) | 3.56 (0.43) | 19 (100) | 3.00 (0.35) | 19 (100) | 2.63 (0.21) | 17 (89) | | 1.24 (0.11) |
|  | Placebo  (N=19) | 16 (84) | 3.63 (0.30) | 19 (100) | 2.84 (0.28) | 17 (89) | 3.18 (0.25) | 16 (84) | | 1.25 (0.11) |
| Cycle 2 | Efgartigimod (N=12) | 10 (83) | 3.10 (0.50) | 12 (100) | 3.08 (0.36) | 12 (100) | 2.92 (0.26) | 11 (92) | | 1.36 (0.15) |
|  | Placebo  (N=14) | 12 (86) | 3.83 (0.32) | 13 (93) | 3.31 (0.40) | 14 (100) | 3.14 (0.25) | 13 (93) | | 1.23 (0.12) |
| QMG | | **n (%)** | **Mean (SE)** (range 1-9) | **n (%)** | **Mean (SE)** (range 1-6) | **n (%)** | **Mean (SE)** (range 1-21) | | **n (%)** | **Mean (SE)** (range 1-3) |
| Cycle 1 | Efgartigimod (N=19) | 19 (100) | 4.37 (0.53) | 12 (63) | 1.67 (0.22) | 19 (100) | 10.79 (0.54) | | 5 (26) | 1.60 (0.24) |
|  | Placebo  (N=19) | 18 (95) | 4.33 (0.44) | 14 (74) | 1.93 (0.25) | 19 (100) | 10.47 (0.73) | | 6 (32) | 1.50 (0.34) |
| Cycle 2 | Efgartigimod (N=12) | 11 (92) | 4.73 (0.75) | 6 (50) | 2.00 (0.45) | 12 (100) | 8.83 (1.07) | | 5 (42) | 1.20 (0.20) |
|  | Placebo  (N=14) | 14 (100) | 4.50 (0.48) | 10 (71) | 2.10 (0.38) | 14 (100) | 10.07 (0.82) | | 5 (36) | 1.40 (0.40) |

^†^Only participants with a baseline score of >0 in each subdomain were included in the analysis. ^‡^ADAPT excluded participants requiring ventilatory assistance and intubation (MGFA Class V), so the maximum possible score in the MG-ADL respiratory subdomain during the ADAPT study was 2 points. MG-ADL, Myasthenia Gravis Activities of Daily Living; MGFA, Myasthenia Gravis Foundation of America; QMG, Quantitative Myasthenia Gravis; SE, standard error.

**Supporting Figure Legends**

**Figure S1.** Percent change from baseline in MG-ADL subdomains over 10 weeks across Cycles 1 and 2 in the overall population. Each cycle consisted of 4 weekly infusions occurring at Weeks 0, 1, 2, and 3 (yellow triangles) of either efgartigimod (10 mg/kg) or matching placebo. AChR-Ab+, acetylcholine receptor antibody–positive; MG-ADL, Myasthenia Gravis Activities of Daily Living; SE, standard error. **P*<0.05 (two-sample *t* test).

**Figure S2.** Percent change from baseline in QMG subdomains over 10 weeks across Cycles 1 and 2 in the overall population. Each cycle consisted of 4 weekly infusions occurring at Weeks 0, 1, 2, and 3 (yellow triangles) of either efgartigimod (10 mg/kg) or matching placebo. QMG, Quantitative Myasthenia Gravis; SE, standard error. **P*<0.05 (two-sample *t* test).

**Figure S3.** Percent change from baseline in MG-ADL subdomains over 10 weeks across Cycles 1 and 2 in the AChR-Ab- population. Each cycle consisted of 4 weekly infusions occurring at Weeks 0, 1, 2, and 3 (yellow triangles) of either efgartigimod (10 mg/kg) or matching placebo. AChR-Ab-, acetylcholine receptor antibody–negative; MG-ADL, Myasthenia Gravis Activities of Daily Living; SE, standard error. **P*<0.05 (two-sample *t* test). ^†^ Differential scaling of y-axis.

**Figure S4.** Percent change from baseline in QMG subdomains over 10 weeks across Cycles 1 and 2 in the AChR-Ab- population. Each cycle consisted of 4 weekly infusions occurring at Weeks 0, 1, 2, and 3 (yellow triangles) of either efgartigimod (10 mg/kg) or matching placebo. AChR-Ab-, acetylcholine receptor antibody–negative; QMG, Quantitative Myasthenia Gravis; SE, standard error. **P*<0.05 (two-sample *t* test). ^†^Differential scaling of y-axis.

**Figure S1.**


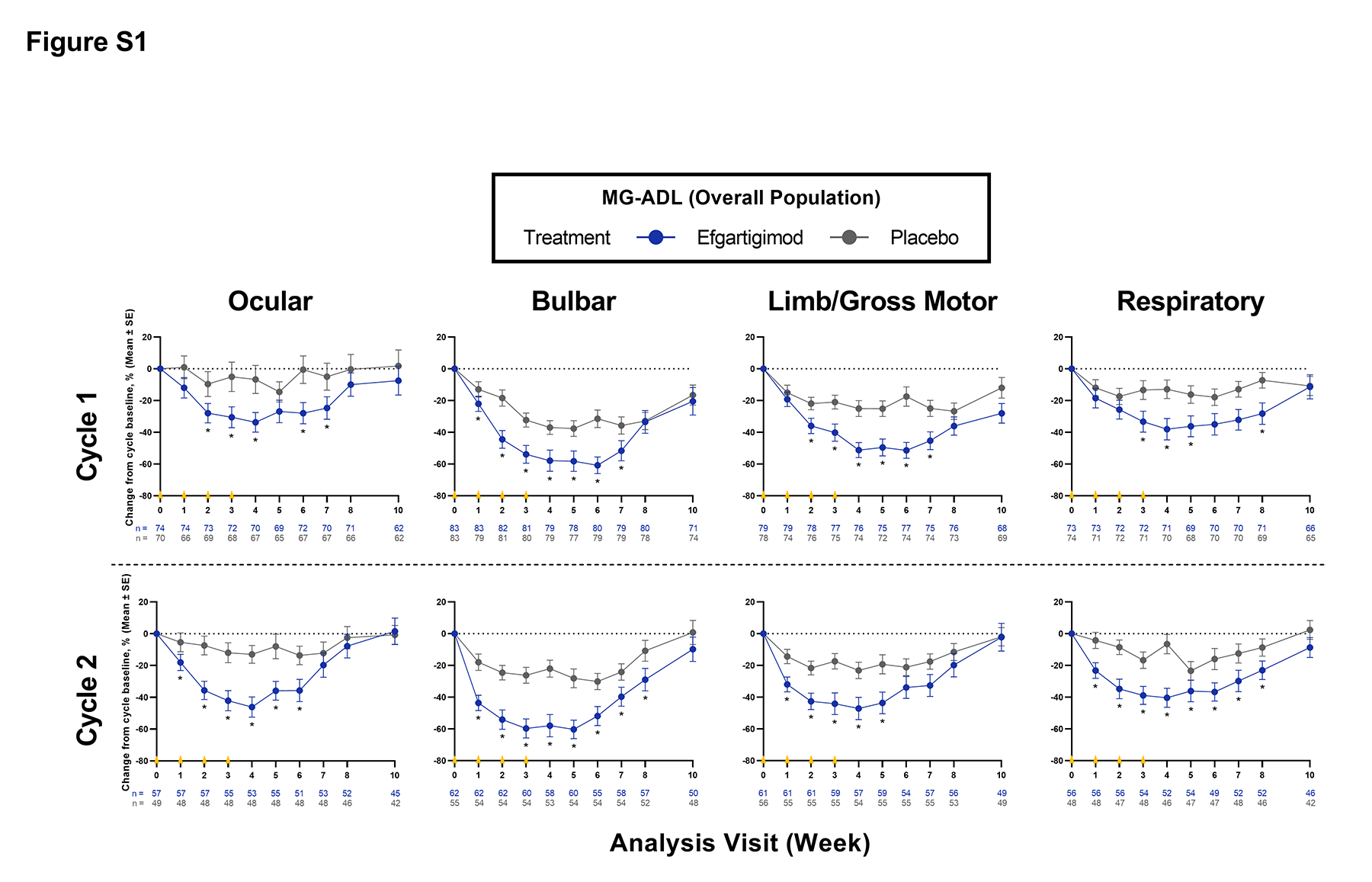


**Figure S2.**


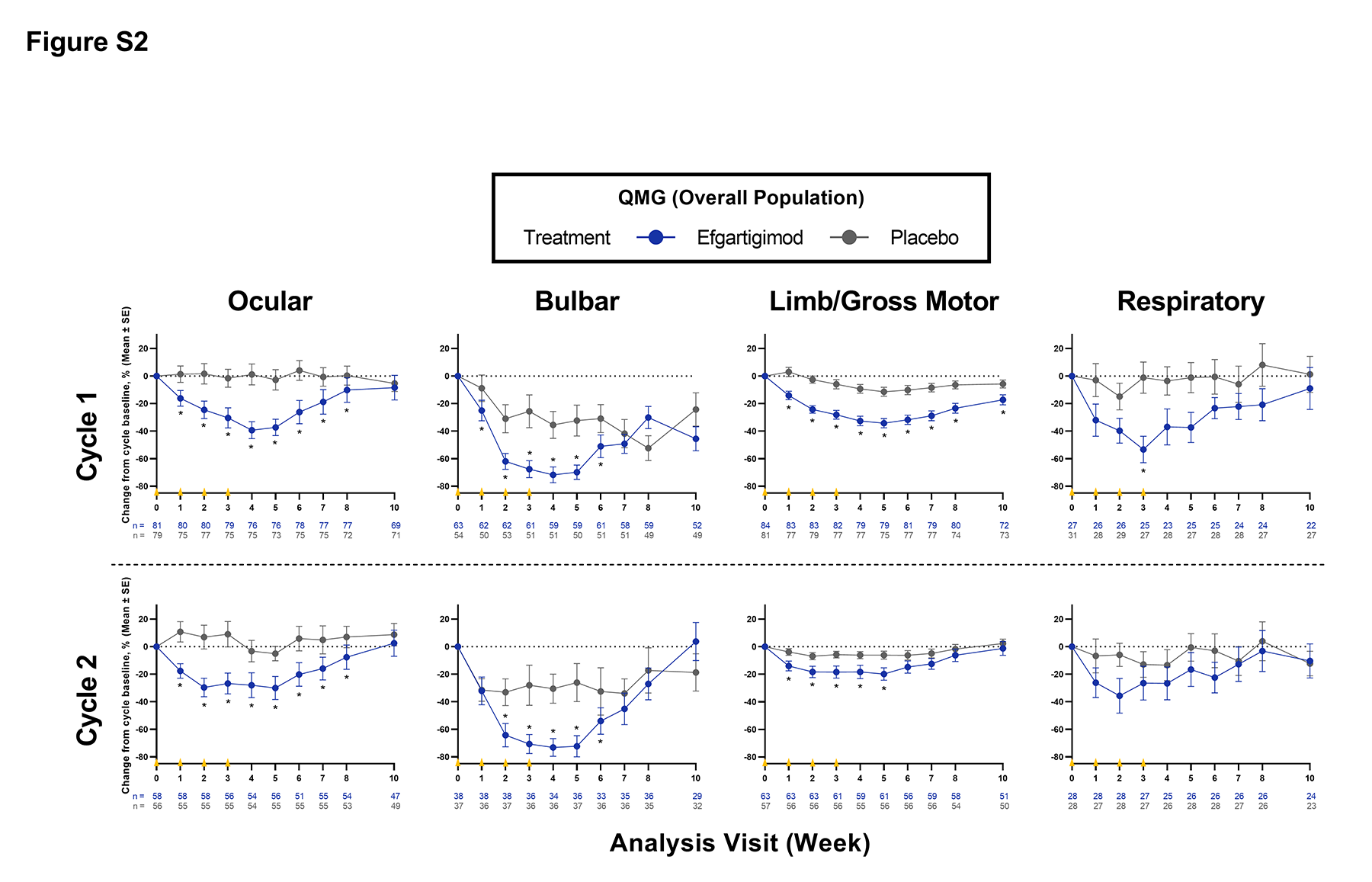


**Figure S3.**


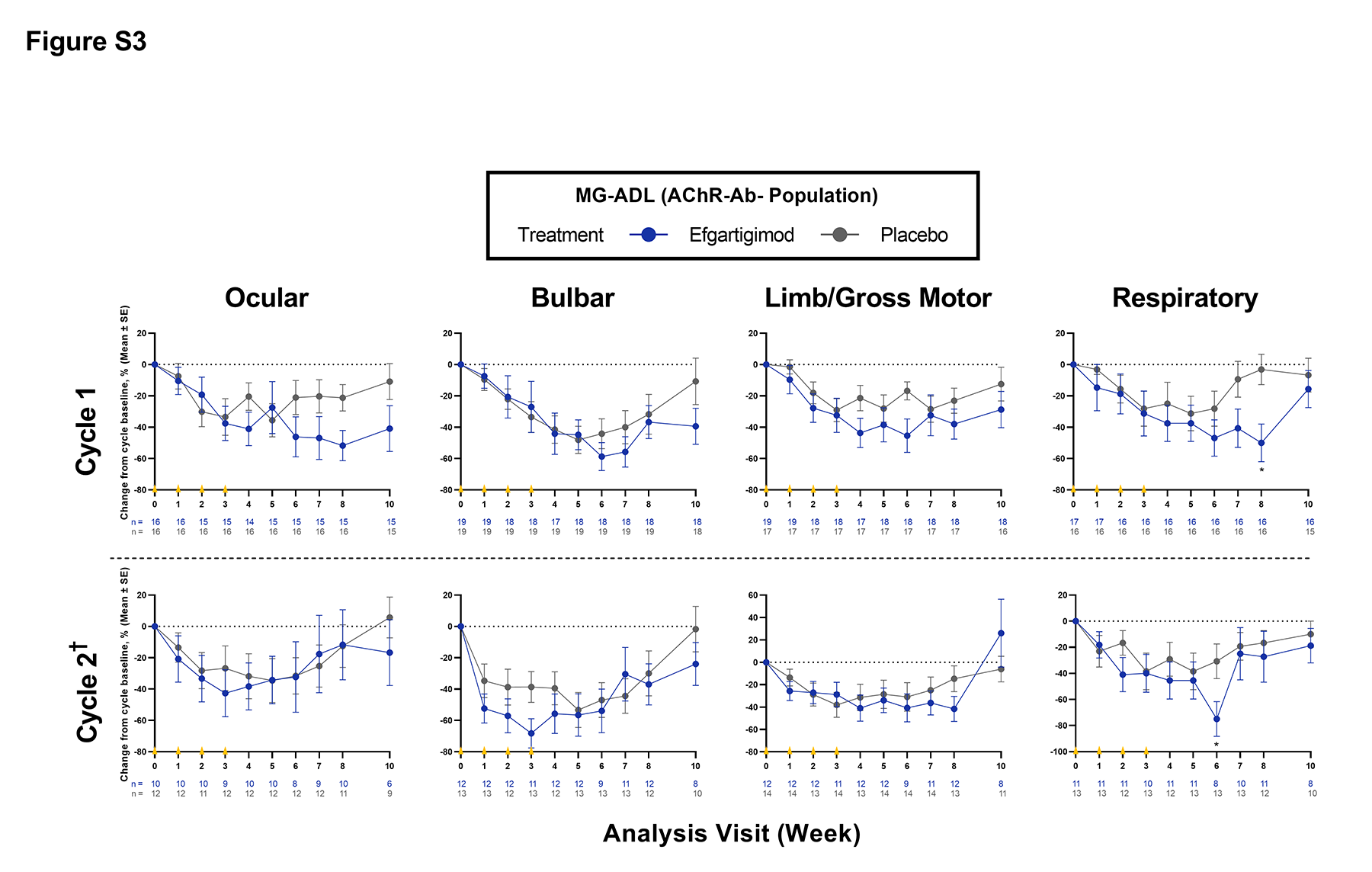


**Figure S4.**


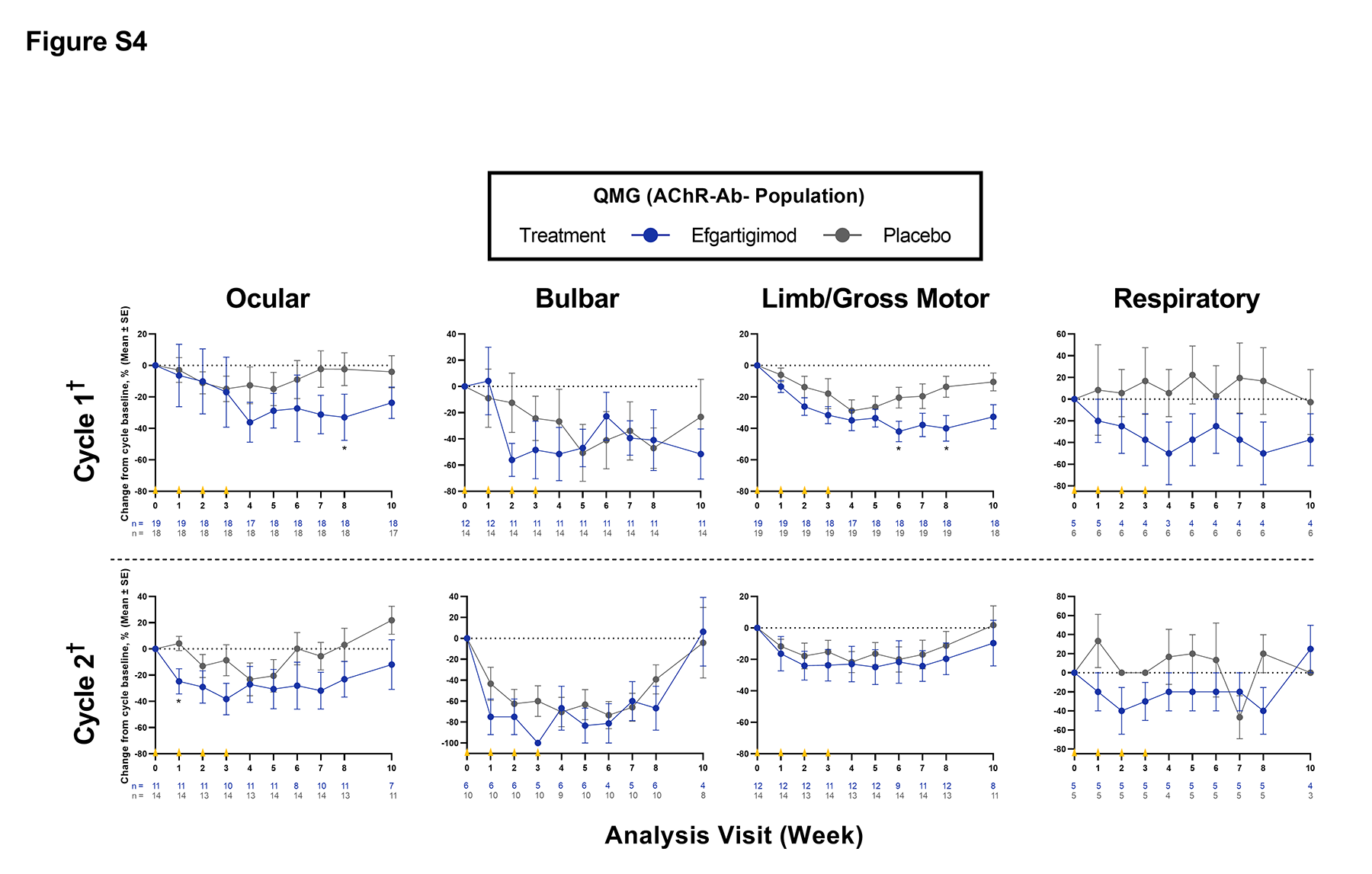

Supplement: Supplementary file 1 — Appendix S1 [file ENE-31-e16098-s006.docx]
