# Supplementary figures and images for: Effect of efgartigimod on muscle group subdomains in participants with generalized myasthenia gravis: post hoc analyses of the phase 3 pivotal ADAPT study
Source: Eur J Neurol. 2023 Oct 16;31(1):e16098. doi: 10.1111/ene.16098 (PMC11235734; doi:10.1111/ene.16098)

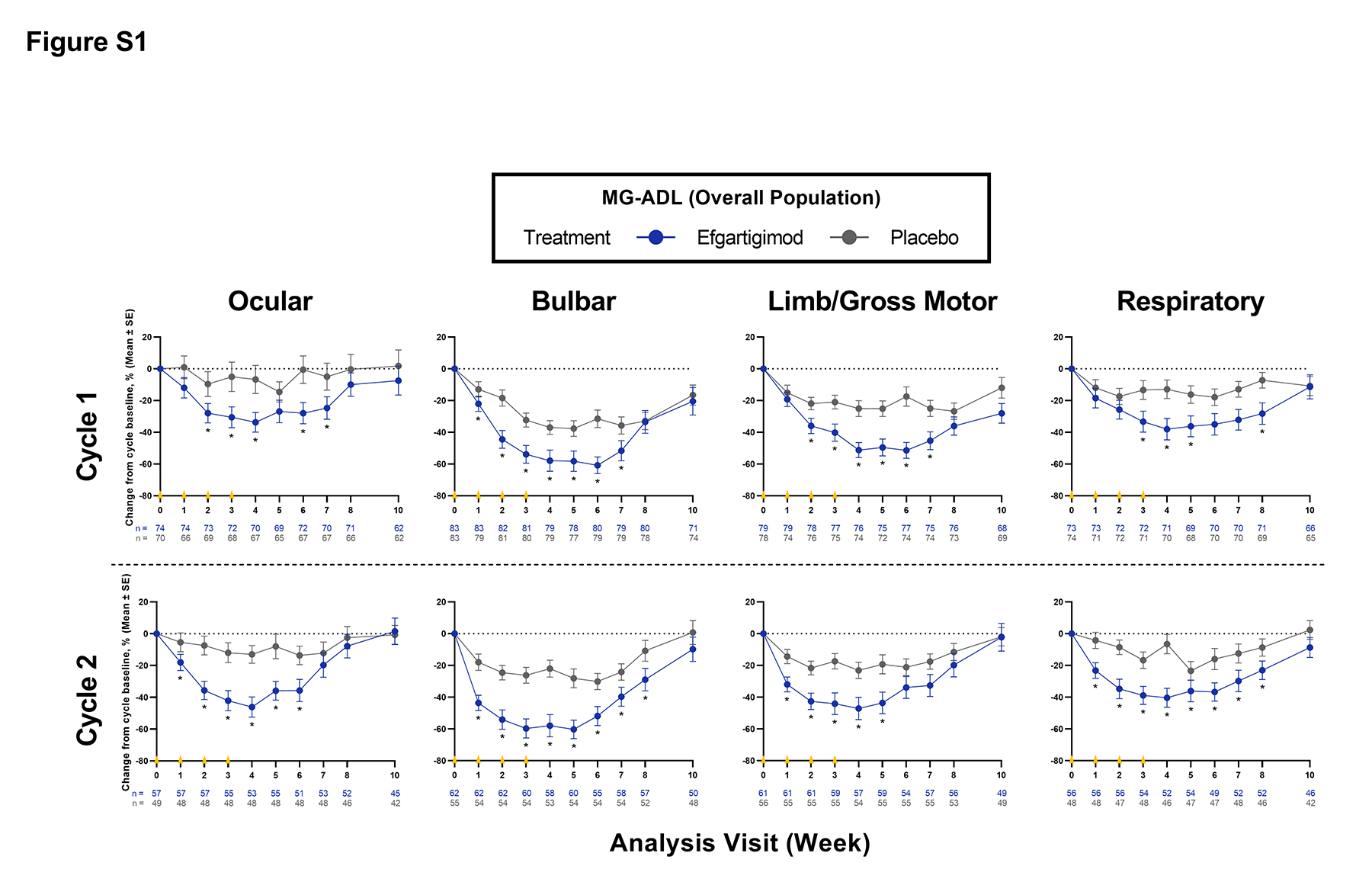

Supplement: Supplementary file 2 — Figure S1 [file ENE-31-e16098-s002.png]

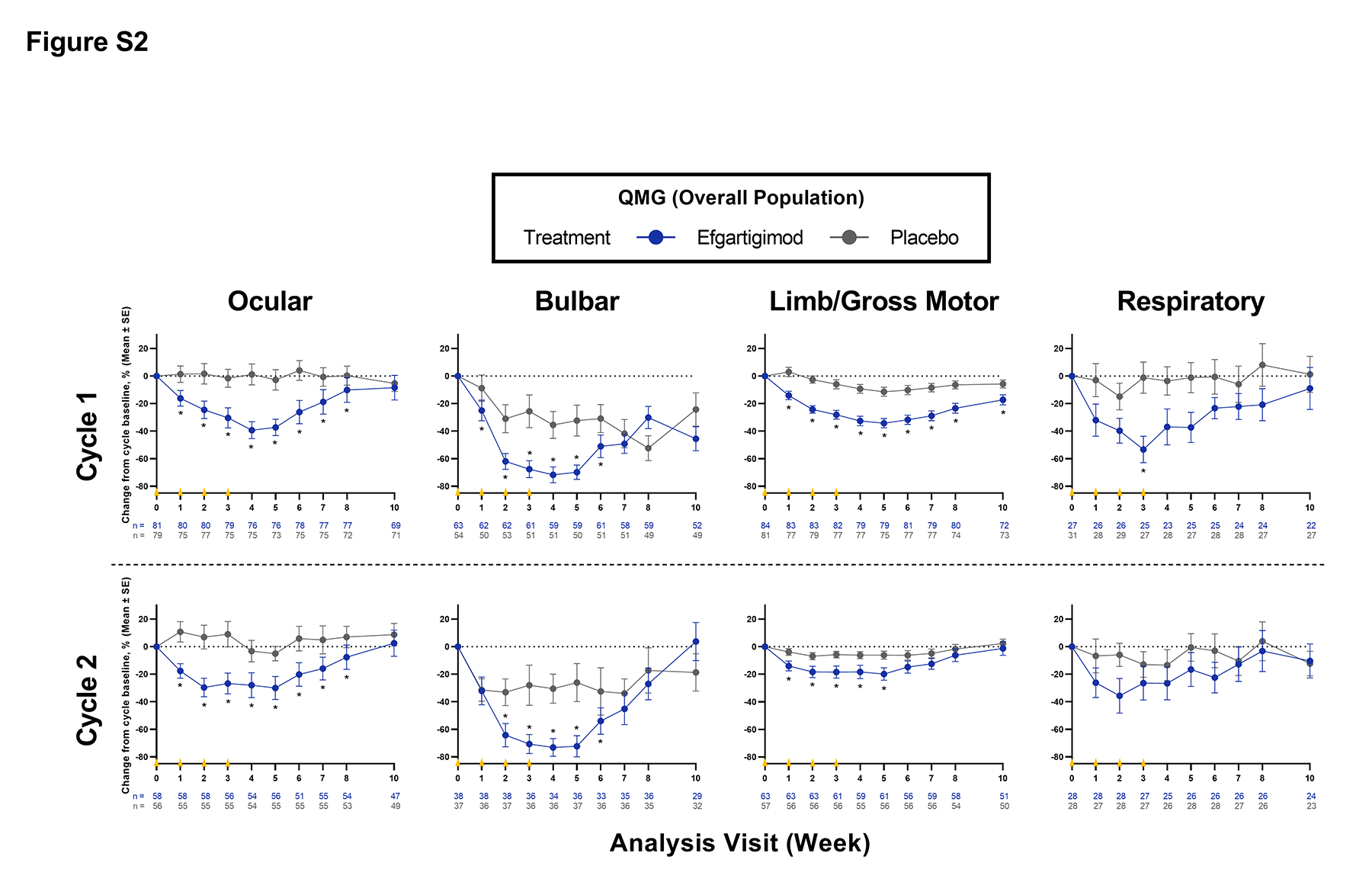

Supplement: Supplementary file 3 — Figure S2 [file ENE-31-e16098-s003.png]

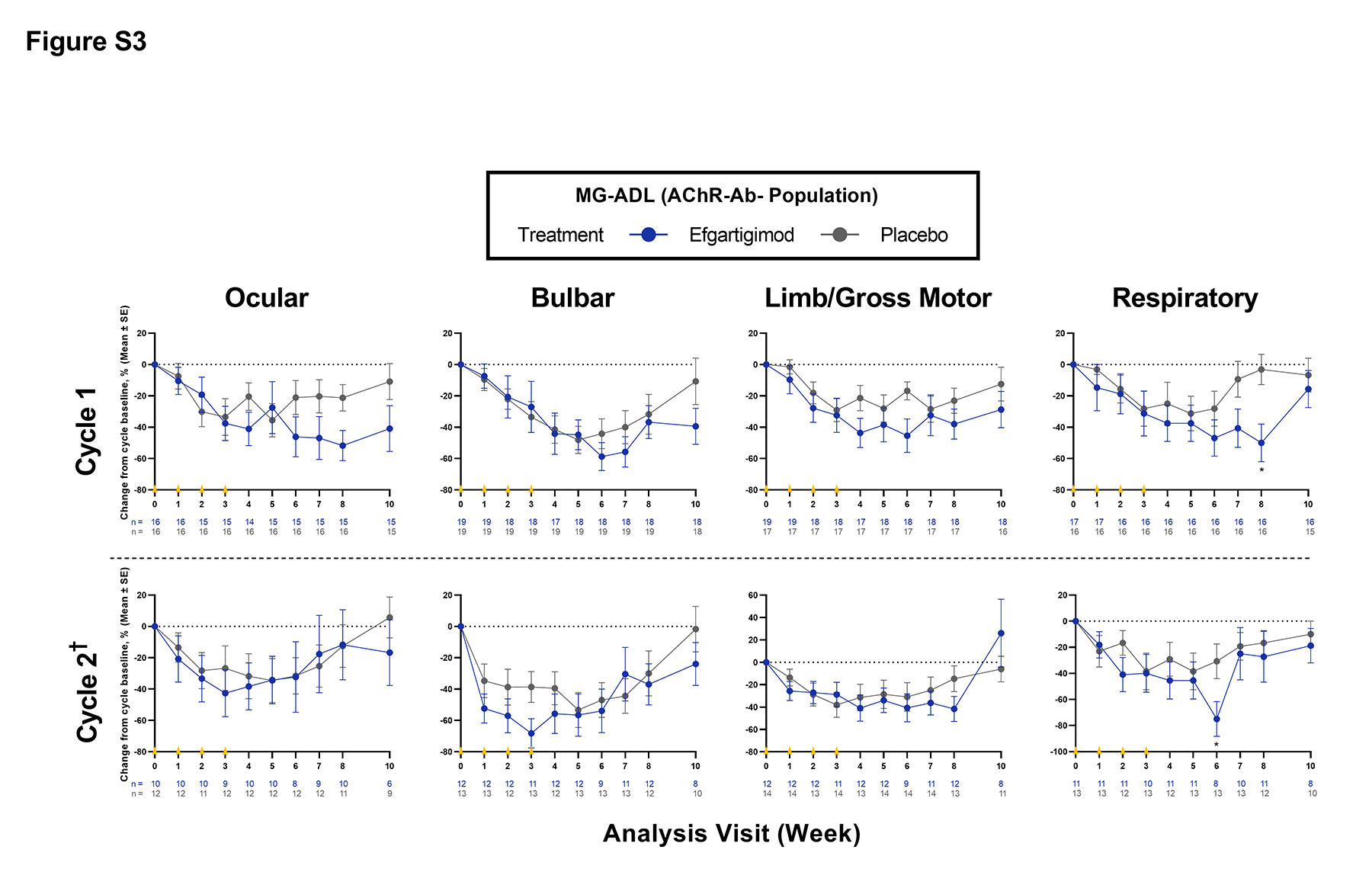

Supplement: Supplementary file 4 — Figure S3 [file ENE-31-e16098-s005.png]

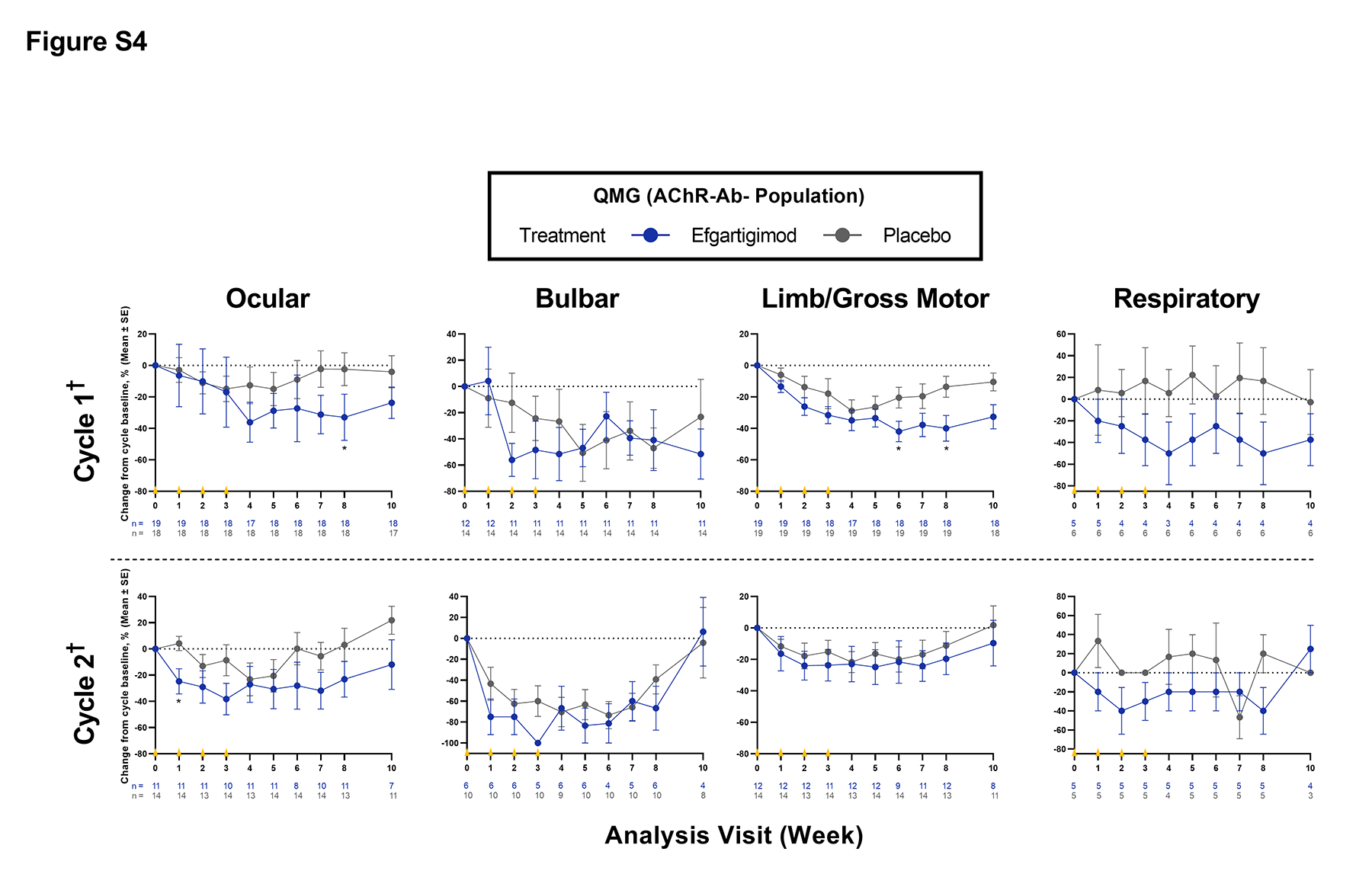

Supplement: Supplementary file 5 — Figure S4 [file ENE-31-e16098-s004.png]
