# Supplementary material for: Effect of efgartigimod on muscle group subdomains in participants with generalized myasthenia gravis: post hoc analyses of the phase 3 pivotal ADAPT study
Source: Eur J Neurol. 2023 Oct 16;31(1):e16098. doi: 10.1111/ene.16098 (PMC11235734; doi:10.1111/ene.16098)
Supplement: Supplementary file 6 — Table S1 [file ENE-31-e16098-s007.docx]

**Supporting Tables**

**Table S1. Disease Activity in MG-ADL and QMG Subdomains at Cycle Baseline in the Overall Population**

|  | Treatment (total N at baseline) | Subdomain^†^ | | | | | | | | |
| --- | --- | --- | --- | --- | --- | --- | --- | --- | --- | --- |
| Assessment |  | **Ocular** | | **Bulbar** | | **Limb/gross motor** | | | **Respiratory** | |
| MG-ADL | | **n (%)** | **Mean (SE)** (range 1-6) | **n (%)** | **Mean (SE)** (range 1-9) | **n (%)** | **Mean (SE)** (range 1-6) | | **n (%)** | **Mean (SE)** (range 1-2^‡^) |
| Cycle 1 | Efgartigimod (N=84) | 74 (88) | 2.84 (0.17) | 83 (99) | 3.12 (0.15) | 79 (94) | 2.73 (0.11) | 73 (87) | | 1.19 (0.05) |
|  | Placebo  (N=83) | 70 (84) | 2.64 (0.15) | 83 (100) | 2.87 (0.13) | 78 (94) | 2.83 (0.12) | 74 (89) | | 1.22 (0.05) |
| Cycle 2 | Efgartigimod (N=63) | 57 (90) | 3.05 (0.18) | 62 (98) | 3.19 (0.18) | 61 (97) | 2.79 (0.13) | 56 (89) | | 1.45 (0.07) |
|  | Placebo  (N=57) | 49 (86) | 3.08 (0.20) | 55 (96) | 3.00 (0.18) | 56 (98) | 2.91 (0.14) | 48 (84) | | 1.25 (0.06) |
| QMG | | **n (%)** | **Mean (SE)** (range 1-9) | **n (%)** | **Mean (SE)** (range 1-6) | **n (%)** | **Mean (SE)** (range 1-21) | | **n (%)** | **Mean (SE)** (range 1-3) |
| Cycle 1 | Efgartigimod (N=84) | 81 (96) | 4.10 (0.24) | 63 (75) | 2.06 (0.13) | 84 (100) | 10.20 (0.33) | | 27 (32) | 1.44 (0.12) |
|  | Placebo  (N=83) | 79 (95) | 3.73 (0.20) | 54 (65) | 1.87 (0.13) | 81 (98) | 10.07 (0.34) | | 31 (37) | 1.48 (0.13) |
| Cycle 2 | Efgartigimod (N=63) | 58 (92) | 4.14 (0.27) | 38 (60) | 2.39 (0.23) | 63 (100) | 9.33 (0.44) | | 28 (44) | 1.29 (0.11) |
|  | Placebo  (N=57) | 56 (98) | 4.09 (0.24) | 37 (65) | 2.08 (0.21) | 57 (100) | 10.51 (0.37) | | 28 (49) | 1.46 (0.15) |

^†^Only participants with a baseline score of >0 in each subdomain were included in the analysis. ^‡^ADAPT excluded participants requiring ventilatory assistance and intubation (MGFA Class V), so the maximum possible score in the MG-ADL respiratory subdomain during the ADAPT study was 2 points. MG-ADL, Myasthenia Gravis Activities of Daily Living; MGFA, Myasthenia Gravis Foundation of America; QMG, Quantitative Myasthenia Gravis; SE, standard error.
