# Supplementary material for: Effect of efgartigimod on muscle group subdomains in participants with generalized myasthenia gravis: post hoc analyses of the phase 3 pivotal ADAPT study
Source: Eur J Neurol. 2023 Oct 16;31(1):e16098. doi: 10.1111/ene.16098 (PMC11235734; doi:10.1111/ene.16098)
Supplement: Supplementary file 7 — Table S2 [file ENE-31-e16098-s001.docx]

**Table S2. Disease Activity in MG-ADL and QMG Subdomains at Cycle Baseline in the AChR-Ab- Population**

|  | Treatment (total N at baseline) | Subdomain^†^ | | | | | | | | |
| --- | --- | --- | --- | --- | --- | --- | --- | --- | --- | --- |
| Assessment |  | **Ocular** | | **Bulbar** | | **Limb/gross motor** | | | **Respiratory** | |
| MG-ADL | | **n (%)** | **Mean (SE)** (range 1-6) | **n (%)** | **Mean (SE)** (range 1-9) | **n (%)** | **Mean (SE)** (range 1-6) | | **n (%)** | **Mean (SE)** (range 1-2^‡^) |
| Cycle 1 | Efgartigimod (N=19) | 16 (84) | 3.56 (0.43) | 19 (100) | 3.00 (0.35) | 19 (100) | 2.63 (0.21) | 17 (89) | | 1.24 (0.11) |
|  | Placebo  (N=19) | 16 (84) | 3.63 (0.30) | 19 (100) | 2.84 (0.28) | 17 (89) | 3.18 (0.25) | 16 (84) | | 1.25 (0.11) |
| Cycle 2 | Efgartigimod (N=12) | 10 (83) | 3.10 (0.50) | 12 (100) | 3.08 (0.36) | 12 (100) | 2.92 (0.26) | 11 (92) | | 1.36 (0.15) |
|  | Placebo  (N=14) | 12 (86) | 3.83 (0.32) | 13 (93) | 3.31 (0.40) | 14 (100) | 3.14 (0.25) | 13 (93) | | 1.23 (0.12) |
| QMG | | **n (%)** | **Mean (SE)** (range 1-9) | **n (%)** | **Mean (SE)** (range 1-6) | **n (%)** | **Mean (SE)** (range 1-21) | | **n (%)** | **Mean (SE)** (range 1-3) |
| Cycle 1 | Efgartigimod (N=19) | 19 (100) | 4.37 (0.53) | 12 (63) | 1.67 (0.22) | 19 (100) | 10.79 (0.54) | | 5 (26) | 1.60 (0.24) |
|  | Placebo  (N=19) | 18 (95) | 4.33 (0.44) | 14 (74) | 1.93 (0.25) | 19 (100) | 10.47 (0.73) | | 6 (32) | 1.50 (0.34) |
| Cycle 2 | Efgartigimod (N=12) | 11 (92) | 4.73 (0.75) | 6 (50) | 2.00 (0.45) | 12 (100) | 8.83 (1.07) | | 5 (42) | 1.20 (0.20) |
|  | Placebo  (N=14) | 14 (100) | 4.50 (0.48) | 10 (71) | 2.10 (0.38) | 14 (100) | 10.07 (0.82) | | 5 (36) | 1.40 (0.40) |

^†^Only participants with a baseline score of >0 in each subdomain were included in the analysis. ^‡^ADAPT excluded participants requiring ventilatory assistance and intubation (MGFA Class V), so the maximum possible score in the MG-ADL respiratory subdomain during the ADAPT study was 2 points. MG-ADL, Myasthenia Gravis Activities of Daily Living; MGFA, Myasthenia Gravis Foundation of America; QMG, Quantitative Myasthenia Gravis; SE, standard error.
